# Tobacco Waste Liquid-Based Organic Fertilizer Particle for Controlled-Release Fulvic Acid and Immobilization of Heavy Metals in Soil

**DOI:** 10.3390/nano12122056

**Published:** 2022-06-15

**Authors:** Dongfang Wang, Jiangshan Li, Xia Yao, Qingchuan Wu, Jing Zhang, Jinghong Ye, He Xu, Zhengyan Wu, Dongqing Cai

**Affiliations:** 1 College of Environmental Science and Engineering, Donghua University, Shanghai 201620, China; dfwang@dhu.edu.cn (D.W.); 2212069@mail.dhu.edu.cn (J.L.); yaoxia16@163.com (X.Y.); zhangjingzj23@163.com (J.Z.); 2191577@mail.dhu.edu.cn (J.Y.); hexu@dhu.edu.cn (H.X.); 2 Key Laboratory of High Magnetic Field and Ion Beam Physical Biology, Hefei Institutes of Physical Science, Chinese Academy of Sciences, Hefei 230031, China; wu1221@mail.ustc.edu.cn (Q.W.); zywu@ipp.ac.cn (Z.W.); 3 Key Laboratory of Environmental Toxicology and Pollution Control Technology of Anhui Province, Hefei Institutes of Physical Science, Chinese Academy of Sciences, Hefei 230031, China

**Keywords:** tobacco waste liquid, CaO, attapulgite, fulvic acid, immobilization

## Abstract

Every year, a large amount of tobacco waste liquid (TWL) is discharged into the environment, resulting in serious pollution for the environment. In this work, a TWL-based particle (OACT) was fabricated by CaO, attapulgite (ATP), and TWL, and, then, OACT was coated by amino silicon oil (ASO) to form OACT@ASO. Therein, OACT@ASO had high controlled-release ability for fulvic acid (FA), because of the nanonetworks structure for ATP and the high content of FA in TWL. The release ratio (RR) of FA from OACT@ASO reached 94% at 75 h in deionized water, and 23% at 32 d in silica sand. Furthermore, the release mechanism of FA from OACT@ASO was consistent with the First-order law. Additionally, OACT@ASO also possessed high immobilization capacity for Cu(II), Cd(II), and Pb(II) (CCP) in soil. Notably, a pot experiment indicated that OACT@ASO could facilitate the growth of pakchoi seedlings and decrease the absorption of CCP by pakchoi seedlings. Thus, this study provides a new kind of organic fertilizer which could not only release FA, but also immobilize CCP in soil.

## 1. Introduction

As we all know, China is the main tobacco producing and consuming country in the world and tobacco plays an important role in our national economy [1]. Every year, there are about 30 thousand tons of tobacco waste liquid (TWL) deposited into the environment, which results in serious pollution for the environment [2]. The processing costs of this TWL are up to 150 dollars/ton, which places huge economic pressure on cigarette factories [3]. In recent years, the state has paid more attention to environmental protection and the discharge standards of wastewater are becoming more and more strict [4,5,6]. Nicotine is the toxic chemical in the TWL and is a serious hazard for the environment and the nervous system [7,8,9,10]. Thus, the treatment and utilization of TWL have become the key bottleneck problems restricting the high-quality development of the tobacco industry. Until now, there have been a few methods focusing on the reuse of TWL. For example, TWL was used to fabricate organic–inorganic compound fertilizer which had the property of water retention, insect pest prevention, and healthy plant growth [11]. TWL could also be converted into hydrochar through the hydrothermal carbonization method and used to remove Cd(II) [12]. However, its application still has certain limitations.

To broaden the application scope, the composition of TWL was analyzed. TWL contains a large amount of nutrients, such as fulvic acid (FA), P_2_O_5_, K, Ca, and some trace elements (Table S1). Furthermore, the amount of FA is much higher than in traditional organic fertilizer [13]. FA, a kind of plant growth regulator, could facilitate the growth, and enhance the production, of crops [14]. Therefore, TWL could be a kind of high-quality organic fertilizer after a certain treatment.

Attapulgite (ATP) is a kind of environment-friendly natural nano clay material containing plenty of nanorods and possessing high adsorption capacity for FA and metal ions, because it has a large specific surface area and nanonetworks structure [15,16,17]. CaO has high hygroscopicity and contains a large amount of calcium, which is beneficial for the growth of crops. ATP/CaO composite, made up of ATP and CaO, possesses a micro/nanoporous structure, which is advantageous to control the release of FA. Since FA has a lot of hydroxyl (-OH) and carboxyl groups (-COOH), ACT, granulated by ATP/CaO and TWL, has a critical role in the immobilization of heavy metal ions [18,19]. In this work, Cu(II), Cd(II), and Pb(II) (CCP) were selected as representative heavy metal ions because of their high toxicity and large amounts [20,21,22]. To improve the stability of the ACT particle, it was coated with amino silicon oil (ASO) to form ACT@ASO.

The objectives of this work were (1) to prepare a TWL-based organic fertilizer particle containing large amounts of FA; (2) to study the release behavior of ACT@ASO for FA in water and silica sand; and (3) to investigate the immobilization capacity of ACT@ASO for CCP. Therefore, it has great potential for application in agriculture and environmental treatment.

## 2. Materials and Methods

### 2.1. Materials

We purchased CaO (98%), Cu(NO_3_)_2_ (99%), Cd(NO_3_)_2_∙4H_2_O (99%), Pb(NO_3_)_2_ (99%), ASO (amino value of 0.5), FA, and other chemicals of analytical grade from Sinopharm Chemical Reagent Company (Shanghai, China). ATP powder (100–200 mesh) was provided by Mingmei Co., Ltd. (Mingguang, China). Tobacco waste liquid (TWL) was obtained from Chongqing Coolbear Technology Co. Ltd. (Chongqing, China). Silica sand (70–120 mesh) was purchased from Nanjing Shuangxiong Filter Material Co. Ltd. (Nanjing, China). Deionized water was used throughout all the experiments, except in the pot experiment. Pakchoi were provided by He Xian Huahe Seed Industry Co. Ltd. (Maanshan, China). Soil and sand (20–50 mesh) were taken from Donghua University (Shanghai, China).

### 2.2. Preparation of OACT@ASO

ATP/CaO composites with different weight ratios (W_ATP_:W_CaO_) were synthesized by mixing ATP and CaO powders evenly. After that, ATP/CaO composites (8 g) were mixed well with TWL (10 g). The optimal ATP/CaO composite with W_ATP_:W_CaO_ = 1:1 was obtained through the water content change of TWL and termed OATP/CaO. Subsequently, the optimal addition amount of OATP/CaO (8 g) in TWL (10 g) was also obtained by the water content change of TWL and designated as OATP/CaO/TWL. OATP/CaO/TWL was granulated with a pelletizer (BY400, Taizhou Changjiang Medicine Machinery Limited Co., Taizhou, China) to get OACT particles with diameters of 1–2 mm. After that, OACT particles (3 g) were immersed in 30 mL of ASO solution for 3 s, and then taken out to a petri dish to get OACT@ASO particles (diameters of 1–2 mm) after air drying.

### 2.3. Release Performance Investigation

#### 2.3.1. Release Performance of FA in Deionized Water from OACT@ASO

OACT@ASO (2.0 g) was put in deionized water (30 mL) at 30 °C. At given intervals, the resulting solution (0.5 mL) was taken out to measure FA concentration with UV-vis spectroscopy. Subsequently, the deionized water (0.5 mL) was supplemented to the system, and then the release ratio (RR) of FA was computed using Equation (1) [23]:

(1) $$\mathrm{RR}\left(\%\right)=\left(\frac{C_{t}\cdot V_{total}+\sum_{0}^{t-1}C_{t}\cdot V_{t}}{m_{0}}\right)\times 100\%$$

where *m*_0_ (mg) represents the initial loading amount, *C_t_* (mg/mL) and *V_t_* (0.5 mL) refers to the FA concentration and solution volume at time *t*, and *V_total_* (30 mL) refers to the total solution volume.

#### 2.3.2. The Investigation of FA Release Kinetics

The First-order, Ritger-peppas, and Parabolic Diffusion models were used to study FA release kinetics in deionized water from OACT@ASO, and these models were shown by Equations (2)–(4) [24]:

ln(1 − M_t_/M_∞_) = −kt (2)

M_t_/M_∞_ = kt^n^ (3)

(M_t_/M_∞_)/t = kt^1/2^ + b (4)

where k represents the kinetic constant, M_t_/M_∞_ refers to the RR (%) of the FA at time t, b refers to a constant, and n represents the release exponent: Fickian diffusion (n < 0.43) and non-Fickian diffusion (0.43 < n < 1.0).

#### 2.3.3. Release Behavior of FA from OACT@ASO in Silica Sand

OACT@ASO (5.0 g) was added to 200 g of silica sand at room temperature. At certain intervals, the resulting silica sand (1 g) was taken out, and then deionized water (5 mL) was added to the silica sand. FA concentration in the supernatant was measured after 1 h with shaking for 160 rpm. Subsequently, 1 g of clean silica sand was supplemented to the system and mixed well with the resulting silica sand.

### 2.4. Leaching Behavior Investigation of OACT@ASO for CCP in the Sand-Soil Mixture

The soil (20–50 mesh) and dry sand (20–50 mesh) were mixed evenly with a weight ratio (W_soil_:W_sand_ = 3:7), and then 30 g of sand-soil mixture (SSM) was added to an injection syringe (60 mL). After that, OACT@ASO, with different weights, were placed on the top of the SSM. Subsequently, the SSM (5 g) was put on the top of the OACT@ASO, and then 40 mL of CCP mixed solution (pH 5.7, 20 mg/L respectively) was placed from the top of the system. Finally, the leachate was collected and its Cu, Cd, and Pb concentrations were detected.

### 2.5. Pot Experiment

Soil (400 g) was put in a trapezoidal pot [length of bottom (7.3 cm) and top (10 cm), and height (8.5 cm)], and then OACT@ASO (5 g) was placed in the top of the soil. After that, 4 pakchoi seedlings (height of 2–3 cm and diameter of 0.1–0.2 cm) were put evenly in the pot, and, then, soil (50 g) was put on top of the pakchoi. Subsequently, the pot was put in a greenhouse (20 °C) and 30 mL of CCP mixed solution (pH 6.6, 20 mg/L respectively) was added to the pot every 2 days.

### 2.6. Characterizations

The morphology was observed using a scanning electron microscope (SEM; S-4800, Hitachi High-Technologies Co., Hitachi, Japan). The pore volumes of OACT and OACT@ASO were determined by means of a pore analyzer (BELSORP-max, MicrotracBEL, Osaka, Japan) through the Barrett-Joyner-Halenda (BJH) method. The structure and chemical state were analyzed using a Fourier transform infrared (FTIR) spectrometer (NEXUS-670, Nicolet Co., Thermo Fisher Scientific, Waltham, MA, USA) and a TTRIII X-ray diffractometer (XRD; D/max-2550VB/PC, Rigaku Co., Hitachi, Japan). Thermal gravimetric analysis (TGA) and differential thermal analysis (DTA) were studied using a thermogravimetric analyzer (DSCQ2000, TA Co., Newcastle, DE, USA). Fluorescence EEM measurements were conducted using a Fluorescence spectrophotometer (F-7000, Hitachi High-Technologies Co., Hitachi, Japan). The CCP concentrations were measured by an inductively coupled plasma-optical emission spectrometer (ICP-OES; ICAP7200, Thermo Fisher Scientific, Waltham, MA, USA). The concentration of FA was detected on a UV-vis spectrophotometer (UV-1900i, Shimadzu Corp., Kyoto, Japan) at 282 nm.

## 3. Results and Discussion

### 3.1. Effect of ATP/CaO on the Water Content of TWL

**Figure 1:**
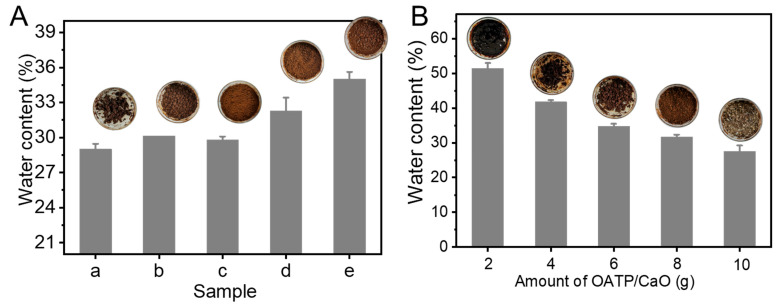
Influence on water content of TWL of different samples at 30 °C: (**A**) ATP/CaO (8.0 g) with different W_ATP_:W_CaO_ of (a) 0:1, (b) 1:3, (c) 1:1, (d) 3:1, and (e) 1:0; (**B**) OATP/CaO with different amounts.

The influence of ATP/CaO on the water content of TWL was studied. Figure 1A shows that the water content of TWL increased slowly before the weight ratio (W_ATP_:W_CaO_) of 1:1 for ATP/CaO, and, then, the water content of TWL increased significantly after the weight ratio (W_ATP_:W_CaO_) of 1:1 for ATP/CaO. This result indicated that CaO could decrease the water content of TWL, which was probably because of the Equation (1). Considering the water content of TWL and cost of ATP (~90 dollars/ton) and CaO (~150 dollars/ton), the optimal weight ratio (W_ATP_:W_CaO_) for ATP/CaO was 1:1 and named as OATP/CaO. After that, the additional amount of OATP/CaO in TWL was also investigated. Figure 1B indicates that the water content of TWL decreased gradually with an increase in the amount of OATP/CaO. Considering the water content and cost performance, 8 g was chosen as the optimal amount for OATP/CaO.

CaO + H_2_O = Ca(OH)_2_ (5)

### 3.2. Release Performance Investigation of FA from OACT@ASO

**Figure 2:**
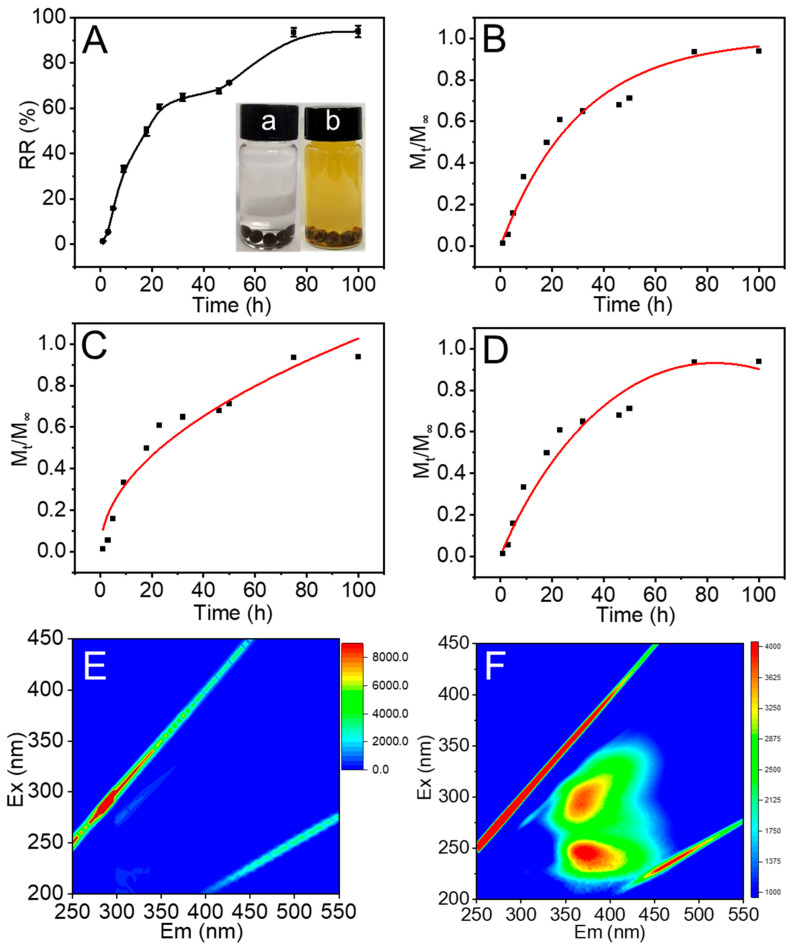
(**A**) The RR of FA from OACT@ASO in water at 30 °C with inset of the digital photographs (a) before and (b) after FA release. Plots of different kinetic models for the release of FA from OACT@ASO: (**B**) First-order, (**C**) Ritger-peppas, and (**D**) Parabolic Diffusion. Three-dimensional fluorescence spectra of (**E**) deionized water and (**F**) the release aqueous solution of OACT@ASO.

Subsequently, the release behavior of FA from OACT@ASO in deionized water was investigated and the concentration of FA was quantified according to Figure S1. The RR of FA improved with time and reached 94% at 75 h, indicating that OACT@ASO possessed a high release efficiency for FA (Figure 2A). Additionally, the release kinetic models were applied to illustrate the release mechanism of FA from OACT@ASO. As shown in Figure 2B–D and Table 1, the release behavior of FA from OACT@ASO was better accorded with the First-order model, which contributed to the higher value of R^2^ (0.9659). These results indicated that the release mechanism of FA from OACT@ASO was consistent with the First-order law. Thus, the release rate of FA was concentration dependent and the FA was proportional to the FA amount released by the unit time diminishment [25]. Meanwhile, Figure 2E,F elucidate that FA could be found at shorter excitation wavelengths (<250 nm) and longer emission wavelengths (>350 nm) [26].

**Figure 3:**
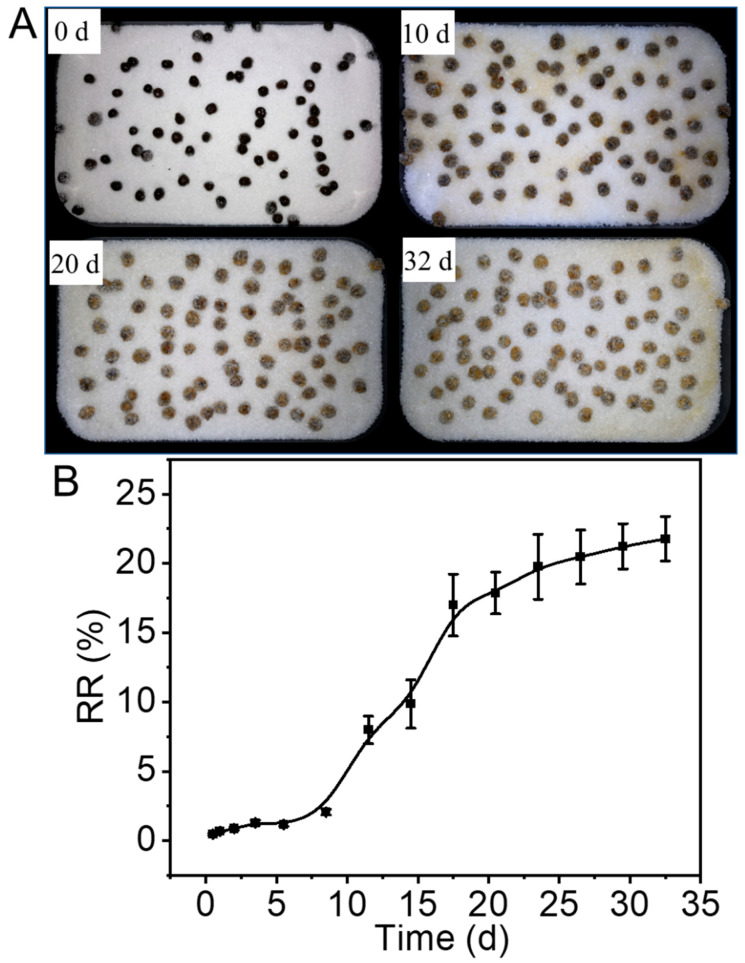
(**A**) Digital photographs of OACT@ASO on silica sand and (**B**) RR of FA from OACT@ASO in silica sand at 30 °C.

The behavior of release of FA from OACT@ASO in silica sand was also investigated. Figure 3A,B indicate that the RR of FA increased significantly before 20 d, then increased slowly after 20 d, and reached 23% at day 32. These results illustrated that the RR of FA in silica soil was lower than in deionized water (94%), probably because the water content of the soil was low. Therefore, OACT@ASO had strong capacity to control FA release in the soil and could be a promising controlled-release fertilizer.

### 3.3. Leaching Behavior Investigation in the SSM

**Figure 4:**
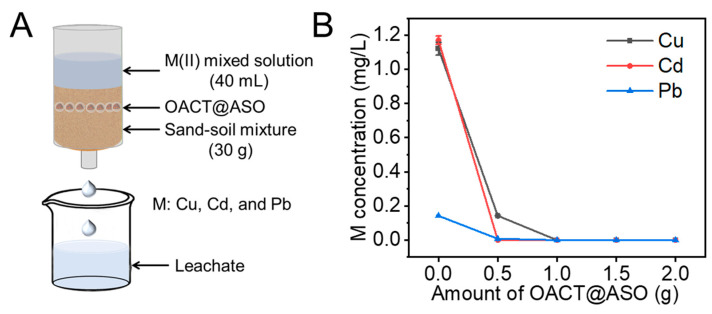
(**A**) Schematic diagram of leaching system. (**B**) Effect of OACT@ASO amount on Cu, Cd, and Pb concentrations in the leachate.

Due to the high solubility, CCP migrated easily and caused wide pollution in the soil [27,28,29]. Thus, it was necessary to decrease the migration amounts of CCP. The effect of OACT@ASO with different amounts on the leaching loss of CCP was investigated. With the increase of OACT@ASO amount, the concentrations of Cu, Cd, and Pb obviously decreased and were nearly zero (Figure 4A,B). Therefore, OACT@ASO possessed high immobilization capacity for CCP and could be a promising Cu(II)/Cd(II)/Pb(II)-containing soil remediation agent.

### 3.4. Mechanism Study

**Figure 5:**
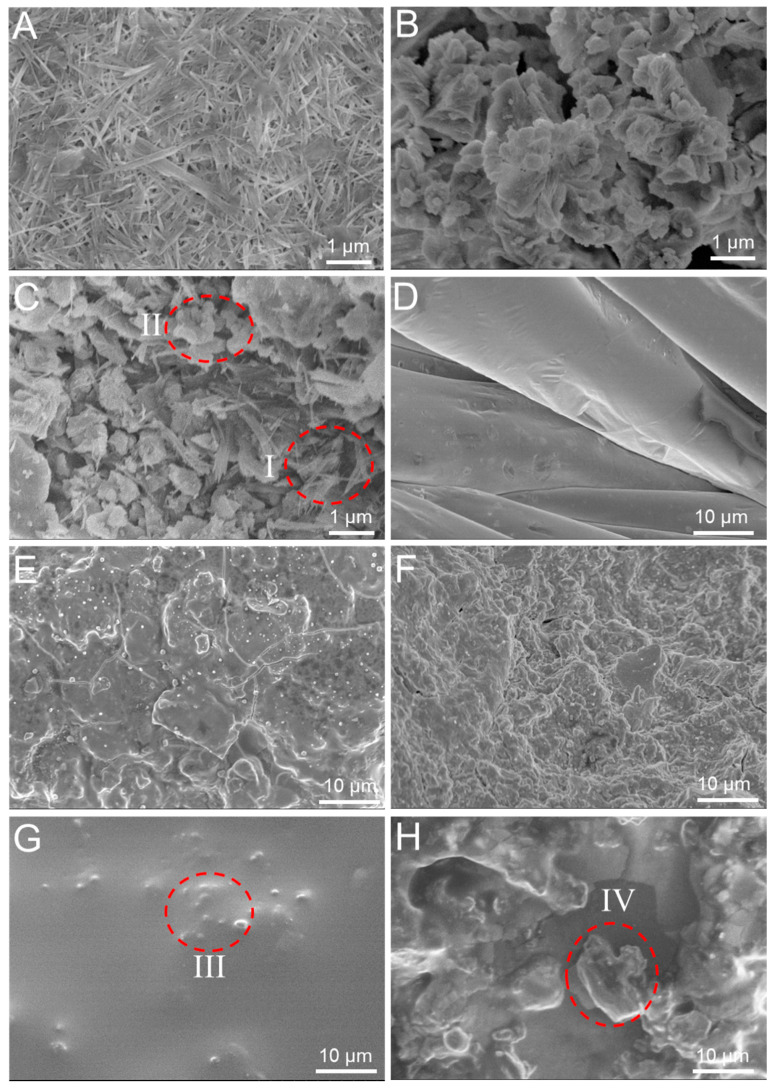
SEM images of (**A**) ATP, (**B**) CaO, (**C**) OATP/CaO, (**D**) TWL, (**E**) surface and (**F**) interior of OACT, (**G**) surface and (**H**) interior of OACT@ASO. Regions I and II note ATP and CaO. Regions III and IV note ASO.

Additionally, to elucidate the release mechanism of FA from OACT@ASO, the morphologies of the OACT@ASO systems were observed. Figure 5A indicates that ATP with nanonetworks structure possessed a large number of nanorods, which was beneficial for the loading of FA in the pores. As shown in Figure 5B, CaO had a rough surface and plenty of micro-nano pores, and, thus, it was favorable for the loading of ATP and FA. When CaO was combined with ATP to form OATP/CaO (Figure 5C), a lot of ATP rods (region I) distributed in the surface and micro-nano pores of CaO (region II) to form a nanonetworks structure, which was beneficial for controlling the release of FA. Figure 5E,F illustrate that the surface and interior of OACT was covered by TWL (Figure 5D), and, thus, OATP/CaO served as the framework of OACT. As shown in Figure 5G,H, ASO (regions III and IV) was distributed on the surface and in the interior of OACT@ASO, which was favorable for controlling the release of FA. Additionally, the total pore volume of OACT@ASO (7.6 mm^3^/g) was lower than that of OACT (8.6 mm^3^/g), also elucidating the fact that OACT was coated by ASO successfully (Figure S2).

**Figure 6:**
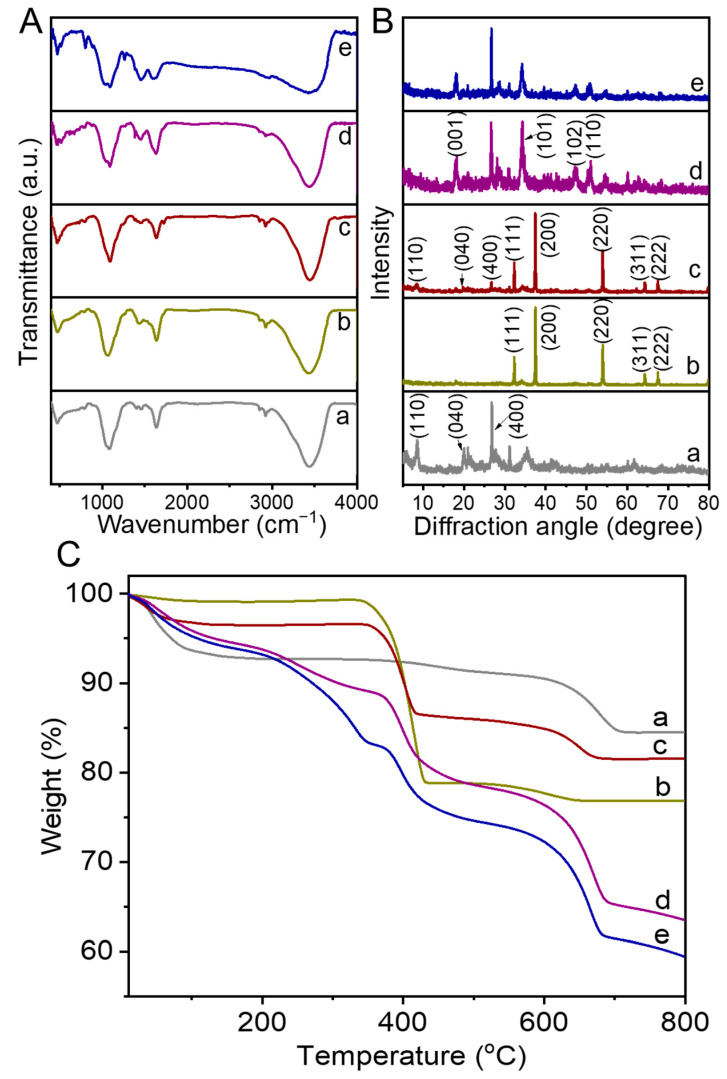
(**A**) FTIR spectra, (**B**) XRD patterns, and (**C**) TGA curves of (a) ATP, (b) CaO, (c) OATP/CaO, (d) OACT, and (e) OACT@ASO.

FTIR measurement was used to illustrate the interactions in the OACT@ASO systems. Figure 6(Aa) shows that ATP had characteristic peaks (stretching vibration of Si-O-Si at 1084 cm^−1^ and translational vibration of -OH at 470 cm^−1^) [30]. After combination of ATP with CaO (Figure 6(Ab)), the peak (stretching vibration of Si-O-Si at 1091 cm^−1^) blue-shifted and the peak (translational vibration of -OH at 469 cm^−1^) slightly red-shifted in the spectrum of OATP/CaO (Figure 6(Ac)). These results illustrated that a hydrogen bond probably existed between ATP and CaO, which could promote the combination. After that, a new peak (C-OH stretching vibration at 1386 cm^−1^) was observed in the spectrum of OACT (Figure 6(Ad)), indicating that TWL combined successfully with OATP/CaO [19]. As shown in Figure 6(Ae), OACT@ASO possessed the characteristic peaks (C-N stretching vibration at 1035 cm^−1^, N-H bending vibration at 1604 cm^−1^, and N-H stretching vibration at 3200–3600 cm^−1^) [24,31]. These results illustrated that OACT was coated successfully by ASO and -NH_2_ existed on the surface of OACT@ASO. Thus, it was beneficial for the chelation of CCP [32]. Furthermore, the peak (C-OH stretching vibration at 1391 cm^−1^) blue-shifted in the spectrum of OACT@ASO, probably due to the existence of a hydrogen bond between OACT (C-OH) and ASO (-NH_2_). Thus, the existence of a hydrogen bond was beneficial for the combination of OACT with ASO, which could improve the stability of OACT@ASO.

In addition, the crystal structures of the OACT@ASO systems were analyzed by XRD measurements. Figure 6(Ba,Bb) show that ATP possessed main peaks corresponding to (110), (040), and (400) and the main diffraction peaks of CaO were (111), (200), (220), (311), and (222) [PDF#37-1497] [30]. In the OATP/CaO pattern (Figure 6(Bc)), the main diffraction peaks of ATP and CaO could be seen and no obvious peak shift was found. Thus, intercalation did not exist between ATP and CaO, and the physical process was the main interaction in OATP/CaO. Figure 6(Bd) illustrates that OACT possessed main peaks corresponding to (001), (101), (102), and (110) for Ca(OH)_2_, which was consistent with reaction (1) [PDF#44-1481]. After coating with ASO, the main OACT peaks could be seen and there was no obvious peak shift in the pattern of OACT@ASO (Figure 6(Be)), indicating that ASO did not change the interplanar crystal spacing of OACT.

The thermal stability of the OACT@ASO systems were studied by TG analysis. Figure 6(Ca–Cc) show that the actual weight percentage of ATP was 63.2% and that of CaO was 36.8% in OATP/CaO. Figure 6(Cd) indicates that the weight loss region (510–690 °C) was probably due to TWL degradation in OACT. Furthermore, the weight loss of OACT@ASO (Figure 6(Ce)) was more obvious than that of OACT at the weight loss region of 510–690 °C, which was likely attributed to ASO decomposition.

### 3.5. Pot Experiment

**Figure 7:**
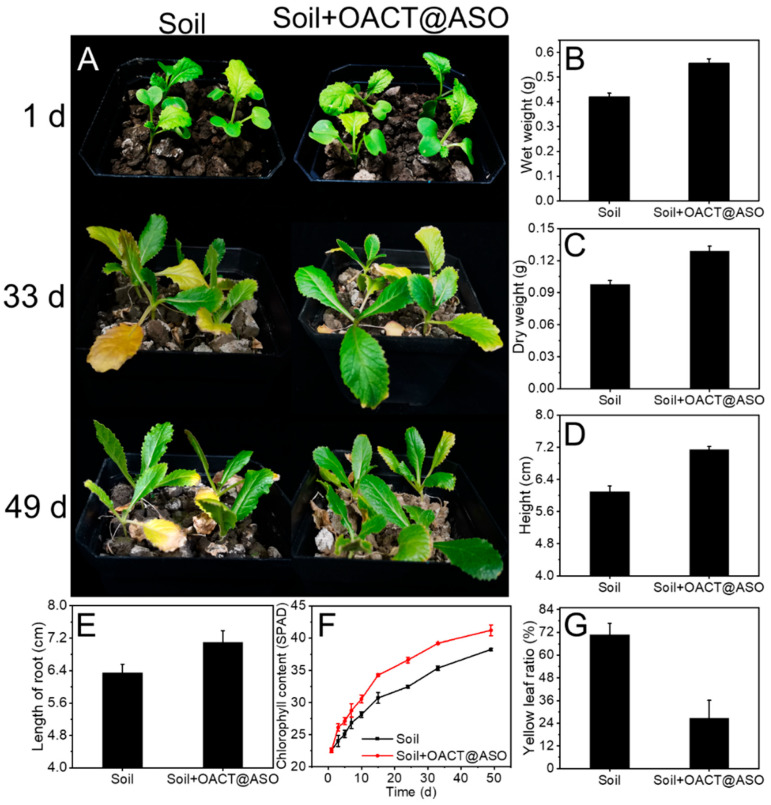
(**A**) Digital photographs of pakchoi seedlings in soil. (**B**–**G**) Wet weight, dry weight, height, length of root, chlorophyll content, and yellow leaf ratio of pakchoi seedlings.

To illustrate the effect of OACT@ASO on the plant and the immobilization capacity of OACT@ASO for Cu, Cd, and Pb, a pot experiment was performed. Figure 7A and Figure S3A show that the growth of pakchoi seedlings with OACT@ASO was obviously better than without OACT@ASO at 33 d and 49 d. In addition, Figure 7B–G and Figure S3B–G indicate that the wet weight, dry weight, height, length of root, and chlorophyll content of pakchoi seedlings with OACT@ASO were higher, and the yellow leaf ratio was lower than without OACT@ASO. These results elucidated that OACT@ASO was beneficial for the growth of pakchoi seedlings. Figure S3H–J show that the Cu, Cd, and Pb amounts in roots, stems and leaves of pakchoi seedlings with OACT@ASO were lower than without OACT@ASO at 49 d. This result illustrated that OACT@ASO possessed a high immobilization capacity for Cu, Cd, and Pb.

A field experiment was carried out to further illustrate the influence of OACT@ASO on pakchoi. OACT@ASO was placed on different distances of the root of pakchoi, and then the growth of pakchoi was investigated. Figure S4 illustrates that the closer OACT@ASO was to the roots the better the plant would grow.

## 4. Conclusions

This work fabricated a kind of TWL-based organic fertilizer particle termed OACT@ASO. The RR of FA from OACT@ASO could reach 94% at 75 h in deionized water and 23% at 32 d in silica sand, respectively. Furthermore, the release mechanism of FA from OACT@ASO was consist with the First-order law. Additionally, a pot experiment and a field experiment indicated that OACT@ASO could facilitate the growth of pakchoi seedlings and decrease the absorption of CCP by pakchoi seedlings. Thus, this study provides a new kind of organic fertilizer particle which could not only release FA, but also immobilize CCP in soil.

## Figures and Tables

**Table 1:** Kinetic fitting parameters of FA from OACT@ASO in water.

	k	n or b	R^2^
First-order	0.0326 ± 0.0020	-	0.9659
Ritger-peppas	0.1050 ± 0.0240	0.4951 ± 0.0573	0.9370
Parabolic Diffusion	−0.0025 ± 0.0003	0.0337 ± 0.0027	0.9476

## Data Availability

Data can be available upon request from the authors.

## Supplementary Materials

The following supporting information can be downloaded at: https://www.mdpi.com/article/10.3390/nano12122056/s1, Figure S1: The relationship between FA and absorbance value; Figure S2: The pore size distributions of OACT and OACT@ASO. Figure S3: Digital photographs of pakchoi seedlings in soil. Wet weight, dry weight, height, length of root, chlorophyll content, and yellow leaf ratio of pakchoi seedlings. Total amounts of Cu, Cd, and Pb in pakchoi seedlings roots, and stems and leaves; Figure S4: Digital photographs of pakchoi at field. Table S1: The composition analysis of tobacco waste liquid.
