# Supplementary material for: Tobacco Waste Liquid-Based Organic Fertilizer Particle for Controlled-Release Fulvic Acid and Immobilization of Heavy Metals in Soil
Source: Nanomaterials (Basel). 2022 Jun 15;12(12):2056. doi: 10.3390/nano12122056 (PMC9227108; doi:10.3390/nano12122056)
Supplement: Supplementary file 1 [file nanomaterials-12-02056-s001.zip › nanomaterials-1749117-supplementary.pdf]

nanomaterials

Supporting Information for:

Tobacco Waste Liquid-Based Organic Fertilizer Particle for Controlled-Release Fulvic Acid and Immobilization of Heavy Metals in Soil

Dongfang Wang [1], Jiangshan Li [1], Xia Yao [1], Qingchuan Wu [2], Jing Zhang [1], Jinghong Ye [1], He Xu [1], Zhengyan Wu [2,3] and Dongqing Cai [1,*]

[1] College of Environmental Science and Engineering, Donghua University, Shanghai 201620, China; dfwang@dhu.edu.cn (D.W.); 2212069@mail.dhu.edu.cn (J.L.); yaoxia16@163.com (X.Y.); zhangjingzj23@163.com (J.Z.); 2191577@mail.dhu.edu.cn (J.Y.); hexu@dhu.edu.cn (H.X.)

[2] Key Laboratory of High Magnetic Field and Ion Beam Physical Biology, Hefei Institutes of Physical Science, Chinese Academy of Sciences, Hefei 230031, China; wu1221@mail.ustc.edu.cn (Q.W.); zywu@ipp.ac.cn (Z.W.)

[3] Key Laboratory of Environmental Toxicology and Pollution Control Technology of Anhui Province, Hefei Institutes of Physical Science, Chinese Academy of Sciences, Hefei 230031, China

* Correspondence: dqcai@dhu.edu.cn

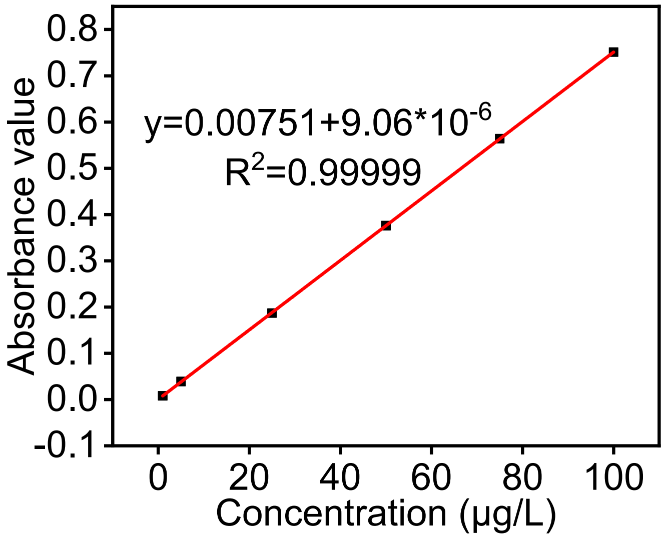

Equation	y = a + b*x
Plot	B
Weight	No Weighting
Intercept	5.90662E-4 ± 5.06896E-4
Slope	0.00751 ± 9.06135E-6
Residual Sum of Squares	2.57929E-6
Pearson's r	1
R-Square (COD)	0.99999
Adj. R-Square	0.99999

Figure S1. The relationship between FA and absorbance value.

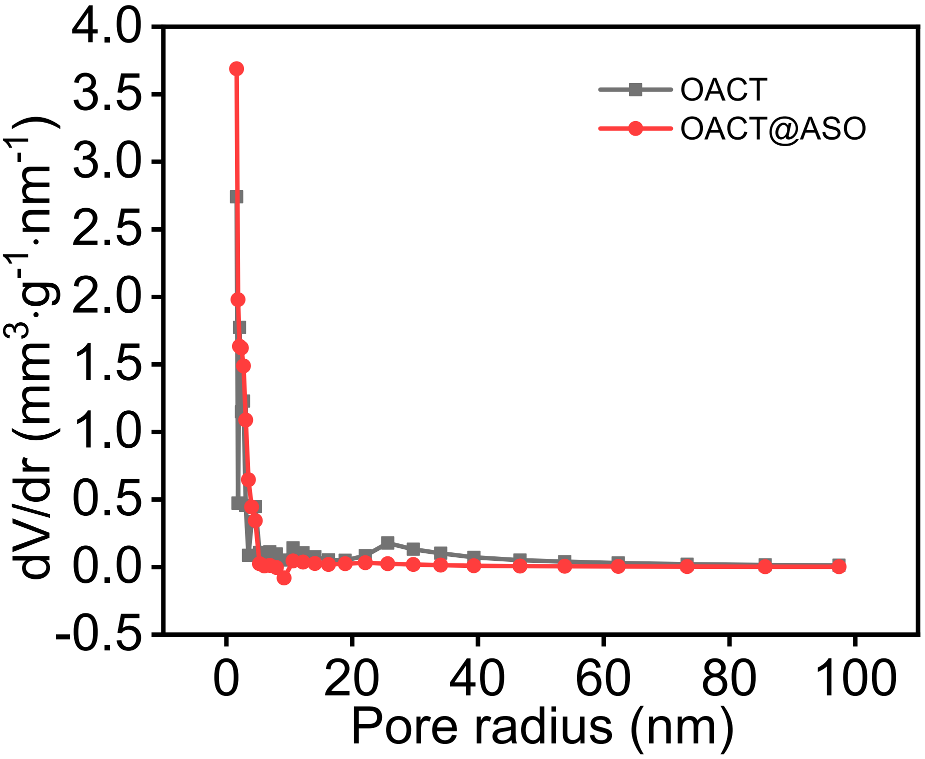

Figure S2. The pore size distributions of OACT and OACT@ASO.

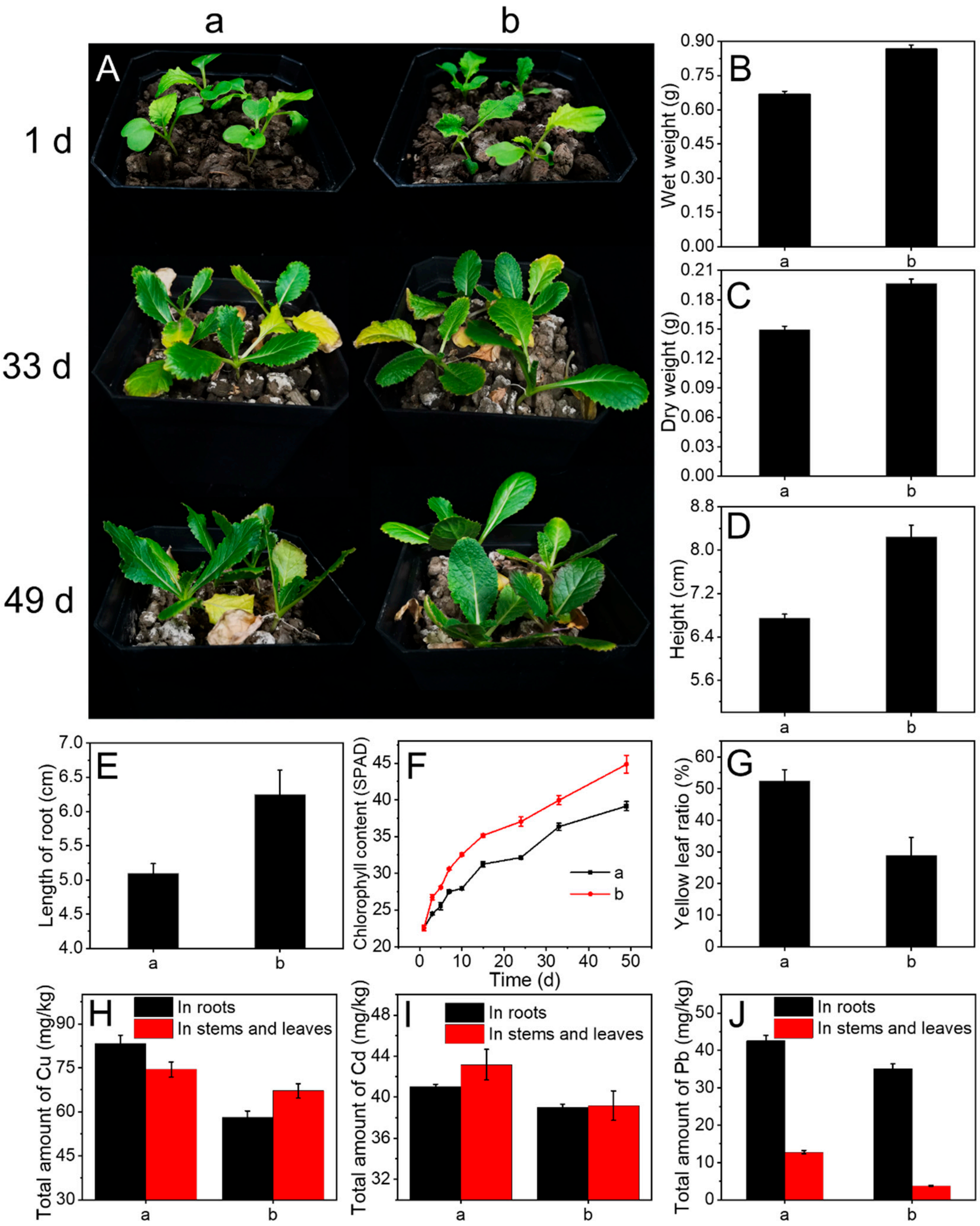

Figure S3. (A) Digital photographs of pakchoi seedlings in soil. (B-G) Wet weight, dry weight, height, length of root, chlorophyll content, and yellow leaf ratio of pakchoi seedlings. (H-J) Total amounts of Cu, Cd, and Pb in pakchoi seedlings roots, and stems and leaves: (a) Soil+Cu(II)/Cd(II)/Pb(II) mixed solution and (b) Soil+Cu(II)/Cd(II)/Pb(II) mixed solution+OACT@ASO.

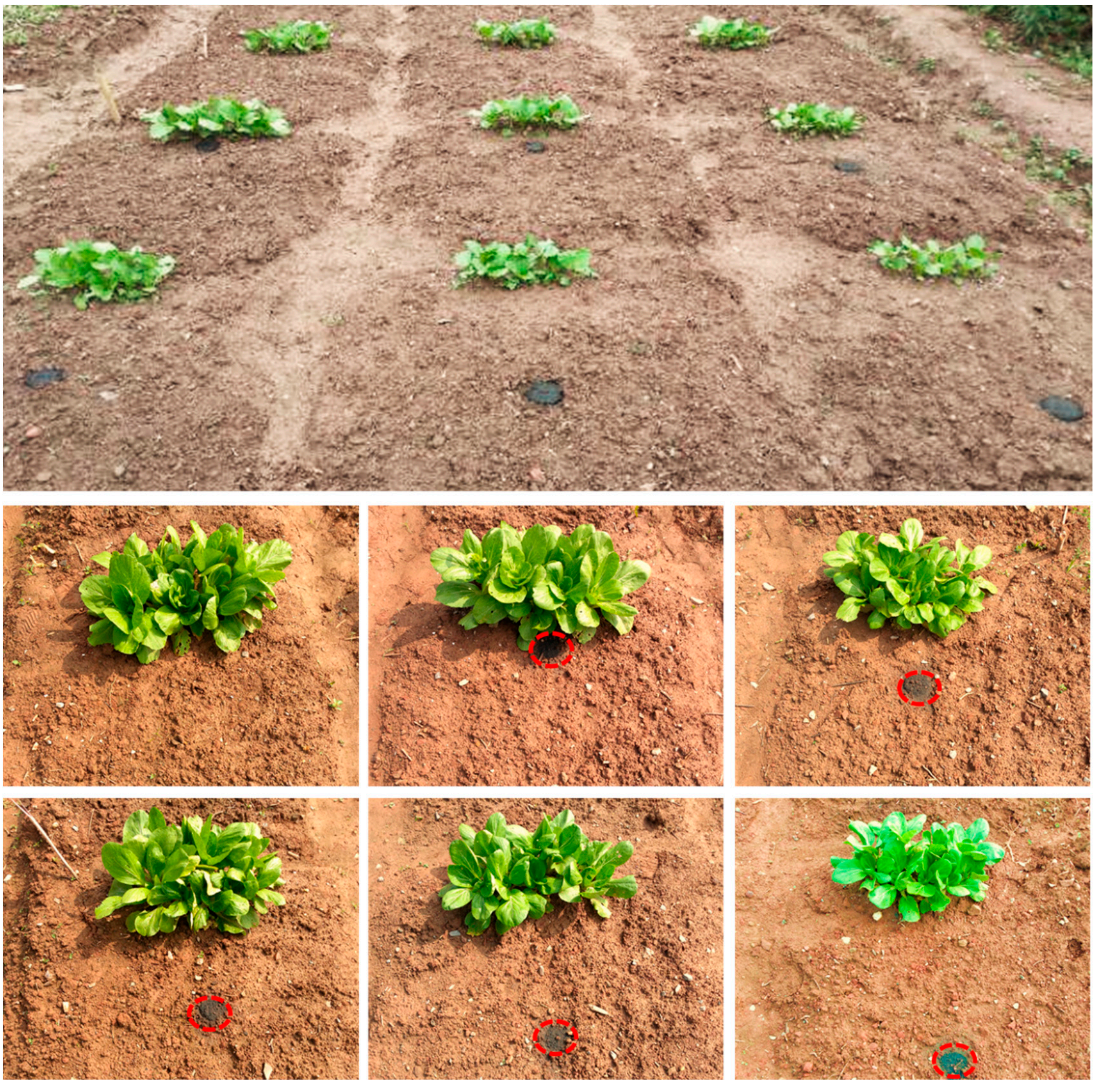

Figure S4. (A) Digital photographs of pakchoi at field.

Table S1. The composition analysis of tobacco waste liquid.

Test Results				
**No**	**Test Item**	**Unit**	**Test Method**	Test Result
1	density	g/mL	NY/T 887-2010	1.19
2	Cu	g/L	NY/T 1974-2010	<0.1
3	Mo	g/L	NY/T 1974-2010	<0.1
4	Mn	g/L	NY/T 1974-2010	<0.1
5	P_2O_5	%	NY/T 1977-2010	0.26
6	S	%	NY/T 1117-2010	0.35
7	B	g/L	NY/T 1974-2010	<0.1
8	Fe	g/L	NY/T 1974-2010	<0.1
9	Mg	mg/kg	NY/T 1117-2010	7.3
10	Zn	g/L	NY/T 1974-2010	<0.1
11	Insoluble Matter	%	NY/T 1973-2010	0.18
12	Total Nitrogen	%	NY/T 1977-2010	0.99
13	Ca	mg/kg	NY/T 1117-2010	9.1
14	pH	-	NY/T 1973-2010	5.07
15	Cl	%	NY/T 1117-2010	2.24
16	Organic Matter	%	NY 525-2012	27.7

17	K	%	NY/T 1977-2010	1.37
18	Humic Acid	%	NY/T 1971-2010	2.37
19	Fulvic Acid	%	Ultraviolet-visible Spectrophotometer	8.70
20	Nicotine Content	%	JY/T 021-1996	0.25
